# Water Pipe Smoking Reduction in the Male Adolescent Students: An Educational Intervention Using Multi-Theory Model

**Published:** 2019-02-05

**Authors:** Saeed Bashirian, Majid Barati, Manoj Sharma, Hamid Abasi, Manoochehr Karami

**Affiliations:** ^1^ Social Determinants of Health Research Center, Hamadan University of Medical Sciences, Hamadan, Iran; ^2^ Behavioral & Environmental Health, School of Public Health, Jackson State University, Jackson, MS, USA; ^3^ Department of Public Health, School of Health, Hamadan University of Medical Sciences, Hamadan, Iran; ^4^ Research Center for Health Sciences, Hamadan University of Medical Sciences, Hamadan, Iran

**Keywords:** Water pipe smoking, Smoking reduction, Health behavior, Students, Adolescent

## Abstract

**Background:** Water pipe smoking (WPS) has increased and is becoming a major leisure pastime among young people in Iran. The aim of this study was to determine of efficacy of an educational intervention based on Multi-Theory Model (MTM) to reduce WPS in the male adolescent students in Iran.

**Study design:** A randomized controlled trial. Methods: Overall, 94 male adolescent students (grades 10, 11) smoked water pipe (WP) in the past month (current WP smokers) were selected, allocated randomly in two groups (47 students in intervention group and 47 students in control group), in two different schools in 2018 in Hamadan City, western Iran. Data were collected utilizing a valid and reliable questionnaire based on MTM constructs and demographic variables. Educational intervention was designed in five 45-min sessions. Two groups were followed-up three-months after completion of intervention. The collected data were analyzed using SPSS 22 software through Chi-square test, independent-sample t-test, paired-samples t-test, and Friedman test.

**Results:** There were significant differences between the mean score of participatory dialogue, behavioral confidence, emotional transformation and practice for change in the intervention group compared with the control group after the intervention (*P*<0.001). In addition, significant reductions in the frequency of WPS (from 14.9% to 4.3%) were observed in the intervention group compared to the control group (*P*<0.001).

**Conclusion:** The developed educational intervention based on MTM constructs was efficacious and can be replicated for effectiveness studies to reduce WPS in the male adolescent students in Iran.

## Introduction

Water pipe (i.e. hookah, narghile, and hubble-bubble) smoking (WPS) has been an old tradition indifferent parts of the world^1^. It has increased globally and threatens to become the second biggest global epidemic after cigarettes^2^. The popularity of water pipe (WP), especially in teens, is increasing who assume it as a social behavior for having fun. WPS contains condensed carbon monoxide, nicotine, tar, and other heavy materials, therefore it poses a greater risk for respiratory diseases, cancers and low birth weight and periodontal disease^3,4^. The prevalence of WPS ranges from 6%-34% in adolescents in the Middle East and 5%-17% among American adolescents,^6^. In Iran, the prevalence of current WPS and ever WPS was 26.3% and 36.4%, respectively^1^, also in another study, WPS prevalence was reported as 17.1% in male high school students^7^. In Iran, 9.7% of students smoked WP in the past month, of which 66.6% were male^1^. Overall, male students are at higher risk of experiencing abusing drugs and smoking than female students^8^.

Adolescents are particularly attracted to the fragrance, to the pipe’s nice appearance, easy accessibility, low cost, less stigma, sensation seeking and greater social acceptance^1,9^. Despite WPS spread and its addictive and harmful potential, studies looking at cessation and treatment options for WP smokers continue to lag behind. Among a representative, population-based sample of adults in Aleppo, Syria, 49% of WP smokers wanted to quit but the quit rates were only 28%^10^. Hence, interventions should be planned and implemented on WP smokers to reduce its use. Such interventions should include building self-efficacy and developing skills for resisting peer pressure^11^. On the other hand, the psychological and social factors are considered important in the first steps of addiction^12^.

**Figure 1:**
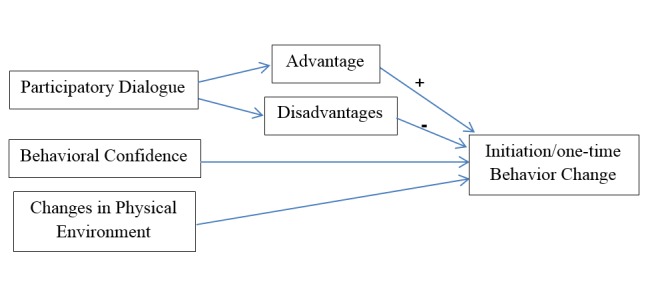
Presents constructs in initiation of health behavior change in multi-theory model

New theories for planning to achieve efficacy and effectiveness of educational programs are needed for developing healthy behaviors^13^. In this context, a new fourth-generation theory has been introduced, multi-theory model (MTM) for health behavior change^14^. The MTM aims to address both initiation and sustenance of health behaviors. MTM has three constructs for initiation: (1) participatory dialogue in which advantages of indulging in a changed behavior outweigh its disadvantages; (2) behavioral confidence, which is somewhat similar to self-efficacy because the ability to perform the behavior is partly internal but also can come from external sources ^14, 15^; (3) changes to the physical environment entail ensuring the tangible resources to support the effort toward changing the behavior^14,15^ (Figure 1).

**Figure 2:**
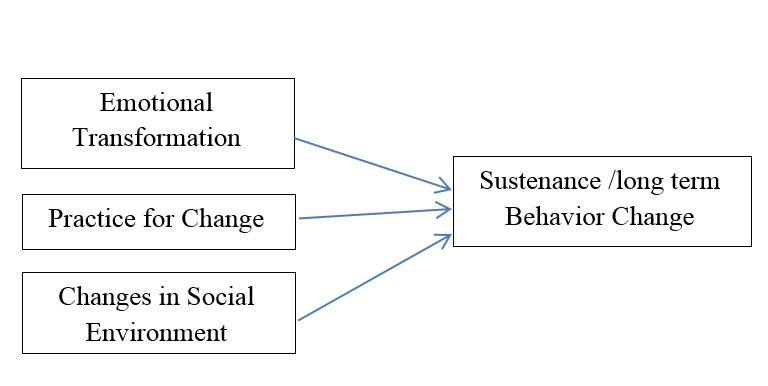
Presents constructs in maintenance of health behavior change in multi-theory model

There are three constructs for sustenance: (1) emotional transformation that relies on the individual’s ability to guide his feelings towards a goal and not succumbing to self-doubt; (2) practice for change consists of the steps or actions; (3) changes in social environment that entail establishing relationships to help goal attainment (Figure 2). This behavior change theory incorporates cognitive, conative, and environmental empirically tested components from existing theories^14^.

There is generally a paucity of studies done on WPS in Iran, especially among high schools ^16^and given that high prevalence of WPS among male adolescent students in Iran, so the purpose of this study was to determine the efficacy of an educational intervention based on MTM to reduce WPS in the male adolescent students in Iran.

## Methods

### Study Population

This randomized controlled trial was conducted among 94 male adolescent students (grades 10, 11) who were studying in technical majors at two schools (interventional school=47, control school= 47) in 2018 in Hamadan City, western Iran. At first, we did a cross-sectional study ^1^ in Hamadan to identify schools and grades with a high prevalence of WPS in the past month (current water pipe smoking). According to the results, 10 and 11 grades had the highest prevalence of WPS in the past month. It was three months interval between two phases of studies (cross-sectional study and interventional study). Considering the power of 90%, Type I error of 0.05 and also Mean ±SD of the behavior of reducing the WPS was 3.26 ±3.2 and 1.6 ±2.46 ^17^, 43 students were calculated. With regard to 15% attrition rate, the sample size was calculated 50 students for each group.

Six students lost in follow-up period, finally data collection were carried out from 94 students (47 students in intervention group and 47 students in the control group). The response rate was 94%. Two schools in two different part of city were assigned as the control group (control school) and the intervention group (intervention school).

The inclusion criteria in this study were: students in 10 and 11 grades, not having the diagnosis of disability or mental and physical diseases, WPS in the past month (current WP smoker), obtaining consent from participants and from their parents. Moreover, the inclusion criteria were 2 absences in training sessions and not available in post-test.

This study was approved by the Ethics Committee of Hamadan University of Medical Sciences (ID: IR.UMSHA.REC.1396.21) and was registered in Iranian Registry of Clinical Trials (IRCT2017042333592N1).

### Instrument

Data collection tool was a researcher-designed questionnaire with two sections. It takes 20-25 min for completing questionnaire by students. The first section was associated with assessment of demographic variables (age, grade, father’s and mother’s occupation, father’s and mother’s education, whether having own room and living status, experienced cigarette smoking, current cigarette smoking) and WPS behaviors (number of WP smoked per month, WPS pattern, having friends having family members who smoke WP, WPS with, WPS place, WPS flavored).

The second section was associated with assessment of MTM constructs. Advantages component of participatory dialogue were assessed with 4 questions, for example “If I reduce WPS, I will have more energy” The choices ranged from completely disagree (=1) to completely agree (=5). The scores for each question were summed to obtain a total possible score for advantages (ranging from 4 to 20 units). Disadvantages component of participatory dialogue were assessed with the four for example “If I reduce WPS, I will not hanging out with friends. The choices ranged from completely disagree (=1) to completely agree (=5). The scores for each question were summed to obtain a total possible score for disadvantages (ranging from 4 to 20 units). We subtracted disadvantages score from advantages scores to calculate participatory dialogue construct score. The second construct, behavioral confidence was assessed with five questions. For example, “I can reduce my WP smoking even if my friends persist with smoking WP”. The choices ranged from completely disagree (=1) to completely agree (=5). The scores for each question were summed to obtain a total possible score for behavioral confidence (ranging from 5 to 25 units).

The environment for which the participants were likely to reduce WP smoking was assessed by six questions. For example, "I can resist attractive coffee houses environment related to WP smoking". The responses ranged from completely disagree (=1) to completely agree (=5). The scores for each question were summed to obtain a total possible score for physical environment (ranging from 6 to 30 units). Emotional transformation was assessed using five questions. For example, “I smoke WP due to enjoyment” Participants selected completely disagree (=1) to completely agree (=5). The scores for each question were summed to obtain a total possible score for emotional transformation (ranging from 5 to 25 units). Practice for change was assessed using five questions. For example, "I can supervise reducing my WP smoking". Participants selected completely disagree (=1) to completely agree (=5). The scores for each question were summed to obtain a total possible score for practice for change (ranging from 5 to 25 units). To assess changes in social environment construct, eight questions were used. For example, "How confident are you that you can get help from close friends to reduce WP smoking?" Participants selected not sure at all (=1) to completely sure (=5). The scores for each question were summed to obtain a total possible score for social environment (ranging from 8 to 40 units). For assessing behavior of WPS in the past month, the question "How many times did you smoke WP in the last month" was designed. Answers included a. Little (less than 15 times) b. Medium (16-30 times) and c. High (more than 30 times).

### Validity and Reliability

After developing the questionnaire, it was pilot tested with 30 students. Their comments on understandability, clarity, and simplicity of items were reviewed and questionnaire edited for clarity (face validity). Validity of the questionnaire was assessed quantitatively. In order to establish content validity, Content Validity Index (CVI) and Content Validity Ratio (CVR) of items were determined by a panel of experts (10 experts in health education). CVR of the instrument was 0.86 as a whole and CVI was 0.91.

Internal consistency reliability of the questionnaire was assessed using Cronbach's alpha which was 0.80 for Participatory Dialogue (advantages 0.80. disadvantages 0.71), behavioral confidence 0.65, changes in physical environment 0.57, emotional transformation 0.74, practice for change: 0.75 and for changes in social environment 0.80.

### Intervention

Designed educational intervention according to the analysis of pre-test results was implemented for the experiment group in five training sessions 45 min for 1 week (Table 1). All of educational sessions were held at the school amphitheater. Pamphlets and a booklet were given to the students after each educational session. First session was focus group discussion that showed photo clips. The focus group discussions were conducted in 10-member groups about advantages and disadvantages of reducing WPS. Second session was focused on students’ behavioral confidence toward reducing WPS. Third session was focused on emotional transformation that influenced students' positive and directing emotions to reduce WPS through showing of video clips. The fourth session was held with the aim of increasing students’ self-monitoring and setting goal of reducing WPS by keeping diary. The fifth session focused on changing the social environment. Moreover, students’ posters designed by health educator about harms effects of WPS, installed in main halls at the school. Parallel to these educational sessions, we created a social network on Telegram and added all of students in intervention group to this channel. This channel included image file of pamphlet, booklet and poster that we designed for them.

**Table 1:** Organization of Educational Sessions in the intervention Group

**Sessions**	**Objectives**	**A summary of topics and activities**
**First**	To facilitate participatory dialogue(Increasing advantages and decreasing perceived disadvantages of reducing WPS)	Group discussion regarding various advantages of reducing WPS and emphasizing on advantages more than disadvantages To show photo clips about advantages of reducing WPS.
**Second**	To facilitate behavioral confidence(increasing self-efficacy and perceived control behavior of reducing WPS)	Role playing about how to persist on presenting WP from peers Video with a media celebrity talked about harmful effects of WPS
**Third**	To facilitate emotional transformation (influences of students' positive and directing emotions to reduce WPS)	Showing video clips harmful effects of WPS Small group discussion about harmful effects of WPS
**Fourth**	To facilitate practice for change(increasing students’ self-monitoring and setting goal of reducing WPS)	Keeping diary notebook for monitoring reducing WPS better Presenting booklets that to direct behavior for reducing WPS goal. To use a role model done self-monitoring in his reducing WPS
**Fifth**	To facilitate change in the social environment (Getting help from friends and family)	Social support from friends and family for reducing WPS. Presenting booklets that to direct behavior for reducing WPS goal. provide emotional and informational support to help reducing WPS in students

Control group received a pamphlet about healthy nutrition during adolescence period. Finally, three months after educational intervention, the questionnaire was completed by both groups again.

### Statistical Analysis

Data were analyzed by SPSS software (ver. 22 (Chicago, IL, USA), paired sample *t*-test (Comparison of mean score of structures in each group), independent-sample t-test (Comparison of the mean score of structures between groups), Chi-square (to compare qualitative variables), Friedman test (testing change in the frequency of WPS in groups) and in this study, *P*<0.05 was considered significant.

## Results

The mean age of the participants was 16.73 (±.642) yr. The mean age at WPS initiation was 13.6 (±2.31) yr. Overall, 38 (80.9%) of participants in the intervention group and 35 (74.5%) in the control group were in the eleventh grade. Regarding the experience with cigarette smoking, 23 (48.9%) of participants in the intervention group and 16 (34%) in the control group had experienced cigarette smoking in the past. Totally, 39 (83%) of participants in the intervention group and 38 (80.9%) in the control group did not smoke cigarettes.

Results of the Chi-square test showed that there were no significant differences between interventional and control groups in age, grade, parents’ education level and occupation, having own room, living (with), experienced cigarette smoking and current cigarette smoking. Table 2 presents demographic characteristics of the students.

**Table 2:** Comparison of the demographic characteristics of the participants in the study

**Demographic variables**	**Intervention group, n=47**		**Control group, n=47**		***P *** **value**
	**Number**	**Percent**	**Number**	**Percent**	
Age (yr)					0.867
15	2	4.3	2	4.3	
16	11	23.4	12	25.4	
17	30	63.8	31	66.0	
18	4	8.5	2	4.3	
High school grade					0.458
Tenth	9	19.1	12	25.5	
Eleventh	38	80.9	35	74.5	
Father's Education					0.428
Illiteracy	1	2.1	0	0.0	
Under the diploma	8	17.0	10	21.3	
Diploma	33	70.3	28	59.6	
College	5	10.6	9	19.1	
Mother's Education					0.336
Illiteracy	0	0.0		12.1	
Under the diploma	9	19.1	10	23.3	
Diploma	32	68.1	25	53.2	
College	6	12.8	11	23.4	
Father’s occupation					0.081
Unemployed	0	0.0	3	6.4	
Worker	10	213	14	29.8	
Self-employed	32	68.3	24	51.1	
Employee	0	0.0	3	6.4	
Retired	5	10.6	3	6.4	
Mother’s occupation					0.370
Housewife	39	83.0	42	89.4	
Employed	8	17.0	5	10.6	
Having own room					0.125
Yes	35	74.5	39	59.6	
No	12	25.5	8	40.4	
Living (with)					0.139
Both parents	41	87.2	45	95.7	
Others	6	12.8	2	4.3	
experienced cigarette smoking					0.143
Yes	23	48.9	16	34.0	
No	24	51.1	31	66.0	
Current cigarette smoking					0.789
Yes	8	17.0	9	19.9	
No	39	83.0	38	80.9	

According to WPS behaviors, 19 (40.4%) of participants in the intervention group and (44.7%) in the control group smoked 15-30 times WP in month. A total of 42 (89.4%) of participants in the intervention group and 40 (85.1%) in the control group had WP user friends. Overall, 36 (76.6%) of participants in the intervention group and 32 (68.1%) in the control group had WP user in the family. There were no significant differences between groups in WPS behaviors (*P*>0.05). Table 3 presents WPS characteristics of the students.

**Table 3:** Comparison of the water pipe smoking behaviors between two intervention and control groups

**Water pipe smoking Behaviors**	**Intervention group, n=47**		**Control group, n=47**		***P*** ** value**
	**Number**	**Percent**	**Number**	**Percent**	
Number of water pipe smoked (times/month)					0.851
Little (<15)	18	38.3	18	38.3	
Medium(15-30)	19	40.4	21	44.7	
More (>30)	10	21.3	8	17.0	
**Water pipe smoking** pattern					0.081
Daily	3	6.5	5	10.6	
Weekly	17	37.0	20	42.6	
Fortnightly	7	15.2	12	25.6	
Every three weeks	3	6.5	5	10.6	
Monthly	16	34.8	5	10.6	
WP user Friends					0.536
Yes	42	89.4	40	85.1	
No	5	10.6	7	14.9	
water pipe user Family					0.356
Yes	11	23.4	15	31.9	
No	36	76.6	32	68.1	
**Water pipe smoking** (with)					0.127
Alone	1	2.1	3	6.4	
Friends	30	63.8	33	70.2	
Family	13	27.7	5	10.6	
Relatives	3	6.4	6	12.8	
**Water pipe smoking** (place)					0.391
At home	15	31.9	19	40.4	
Cafe	32	68.1	28	59.6	
water pipe flavored					0.231
Yes	42	91.3	39	83.0	
No	4	8.7	8	17.0	

Results of the paired sample *t*-test showed that significant increase occurred in the mean scores of participatory dialogue (*P*=0.037), behavioral confidence (*P*<0.001), emotional transformation (*P*<0.001) and practice for change (*P*=0.001) in the intervention group as compared to the control group after intervention. Independent t-test also revealed that there were no significant differences between groups before intervention in the mean scores of participatory dialogue (*P*=0.845), behavioral confidence (*P*=0.920), changes in physical environment (*P*=0.908), emotional transformation (*P*=0.626), practice for change (*P*=0.980) and changes in social environment (*P*=0.434). There were significant differences between groups after intervention in the mean scores of participatory dialogue (*P*=0.008), behavioral confidence (*P*=0.007), emotional transformation (*P*<0.001), practice for change (*P*<0.001) (Table 4).

**Table 4:** Comparison of mean scores of multi -theory model constructs before and 3 months after intervention in the control group and intervention group

**Variabl**	**Intervention group** **( n=47)**		**Control group** **( n=47)**		**Diff. (95% CI)**	***P*** ** value**
**MTM Constructs**	**Mean**	**SD**	**Mean**	**SD**		
Participatory dialogue: Advantages-Disadvantages					
Baseline	1.60	2.66	1.53	4.11	0.15 (-1.36, 1.65)	0.845
Follow-up	2.91	1.76	1.45	4.41	-1.91 (-3.31, -0.51)	0.008
*P* value	0.037		0.108			
Behavioral confidence						
Baseline	19.60	3.45	19.90	4.59	0.10 (-1.53, 1.71)	0.920
Follow-up	22.02	2.81	20.36	3.00	-1.66(-2.85, -0.46)	0.007
P value	0.001		0.070			
Changes in physical environment						
Baseline	19.72	4.06	19.13	4.25	0.10 (-1.72, 1.93)	0.908
Follow-up	19.06	4.64	19.51	4.66	0.74 (-1.22, 2.70)	0.453
P value	0.124		0.080			
Emotional transformation						
Baseline	13.87	3.32	14.26	4.41	0.38 (-1.17, 1.93)	0.626
Follow-up	17.45	3.03	13.03	1.36	-5.83 (-6.90, -4.76)	0.001
P value	0.001		0.053			
Practice for change						
Baseline	18.60	4.02	16.66	3.21	0.1 (-1.78, 1.84)	0.980
Follow-up	21.15	3.30	16.96	2.25	-5.17 (-6.63, -3.70)	0.001
P value	0.001		0.131			
Changes in social environment						
Baseline	16.60	6.15	17.55	5.65	0.96 (-1.46, 3.37)	0.434
Follow-up	17.13	5.92	17.81	2.90	-1.34 (-3.42, 0.74)	0.204
P value	0.706		0.725			

The results of Friedman test showed that there was a significant difference in the frequency of WPS in the intervention group compared to the control group (*P*<0.001) (Table 5).

**Table 5:** Comparison of the number of water pipe smoking in the last month before and after 3 month in the intervention (n=47) and control group (n=47)

**Number of water pipe smoked per month**	**Before**		**After**		***P*** ** value**
	**Number**	**Percent**	**Number**	**Percent**	
Intervention group (times/month)					0.001
None	1	2.1	4	8.5	
Little (<15)	20	42.6	26	55.3	
Medium (15-30)	19	40.4	15	31.9	
More (>30)	7	14.9	2	4.3	
Control group (times/month)					0.848
None	0	0.0	1	2.1	
Little (<15)	23	48.9	23	48.9	
Medium (15-30)	20	42.6	17	36.2	
More (>30)	4	8.5	6	12.8	

## Discussion

The present study was conducted with the aim of determining the effects of an educational intervention based on MTM on reducing WPS among male adolescent students. The results revealed significant changes in participatory dialogue, behavioral confidence, emotional transformation, practice for change constructs and the behavior of reducing WPS in interventional group compared to the control group after intervention.

The participatory dialogue structure in the MTM is derived from the perceived benefits and barriers construct of the Health Belief Model and the decision-making balances of Transtheoretical Model (TTM)^14^, interventions had a significant effect to increase participatory dialogue construct, so that the mean scores of intervention and control group before and after intervention showed a significant difference. By conducting group discussions and emphasizing on perceived benefits of reducing WPS more than perceived barriers of reducing WPS, as well as using photo clip, pamphlet, booklet and reminder on the telegram, it increased the score of participatory dialogue in the intervention group compared to the control. The results of previous studies ^13, 18^ are consistent with present study. The mean score of perceived benefits and perceived barriers in the intervention group was higher than the control group after training session^13^. In addition, the mean score of perceived benefits and barriers increased in the intervention group increased compared to control group after intervention.

The other finding of this study was the increase in the mean score of behavioral confidence construct in the intervention group after educational intervention. Teaching behaviors in a confidence-building manner, or not encouraging use of hookahs in the form of role-play, video clips, and the use of pamphlet influenced enhancing students' self-efficacy. By determining the level of self-efficacy of water pipe smokers, self-efficacy enhancement strategies can be of great importance through the motivation and continuity of the behavior, saying no skill, the increase in self-esteem and self-belief in one's ability to reduce and quit the behavior. Self-efficacy and perceived behavioral control score increased after intervention in the intervention group compared to the control group^19-22^. In the studies mentioned the use of educational techniques and strategies such as role-playing, and the importance of confident behavior change could have a positive impact on the level of self-efficacy in preventing and reducing the WPS.

The other findings of this study were lack of significant effect in the structure of changes in physical environment before and after educational intervention in both intervention and control groups. Changes in the environmental characteristics need to be of health policy-making, legislation and high-level decisions. In fact, the change in the physical environment requires a health promotion from the Ministry of Health and in this study, assessing or encouraging of the environment has been related to the prevalence of WPS. The role of facilitating factors such as availability, price, availability, purchasing power and the ability to increase the prevalence of WPS in the community, especially in young people^23,24^.

The results of this study showed that after the intervention, the mean score of the emotional transformation structure in the intervention group was significantly higher than the control group. The WP smokers are interested in taste, smell and sound of it, so in this study, using video clip, pamphlet and poster emphasized the harmfulness of WPS and its risks to the health changed positive tendencies and emotions associated with WPS and focused on managing feelings directed with the behavior of reducing WPS. The results of previous studies^20,25,26^ are consistent with present study.

The other result of the present study was in the mean score of practice for changing after intervention in the intervention group compared to the control group. Given that the practice for change in MTM includes individual's supervision, self-regulation on changing behavior and focusing on the target's behavior, using the booklet, using of role model had an effect on the target's behavior of reducing WPS. Students who had less self-regulated feelings than other students had a higher chance of starting smoking ^27^. Regarding the lack of study in the field of the role of self-regulation in WPS, using self-regulating process and self-regulating mechanism students can make changes to them and direct them in reaching the goal, so that in person the probability the behavior of reducing water pipe will increase.

In this study, changes in the social environment did not register significant change in the intervention group compared to the control group. The educational methods in this study could not encourage family and friends to reduce the WPS for the student. The role of friends and family members in WPS is important and they can play a preventive and persuasive role in reducing WPS^28^. Another important finding of this study was the behavior of reducing WPS in the intervention group compared to control group after the intervention, even some students quitted WPS. Therefore, MTM is a perfect, rational and comprehensive model that considers all behavioral factors in behavioral change and it could reduce WPS in male adolescent student. The results of various studies^29-33^ also showed a significant decrease in the use of WP in the intervention group compared to the control group.

A limitation of this study was that there was not any study on educational interventions based on the use of MTM to reduce WPS in adolescents, which limited the power of comparing findings and decision making in the field. Because of challenges getting permission to enter the girls’ high schools, the current study was restricted to only male high schools. These findings may therefore not apply to all Iranian adolescents. Due to executive limitations such as the interference of the educational program with classrooms and workshop programs, training sessions were conducted during 1 wk and 5 sessions. Since the method of completing questionnaires and reports on the frequency of WPS was self-report, some participants may not have answered honestly. Future studies must use more objective methods such as measuring the level of blood monoxide to investigate the effect of interventions.

## Conclusion

Use of MTM is effective in increasing the perceived benefits of reducing, enhancing behavioral confidence, managing positive emotions toward reducing WPS and monitoring more effectively the behavior of reducing WPS. Interventions based on MTM also could reduce and quit WPS students. Therefore, in designing and implementing interventional programs using the MTM, the factors that reduce the WPS can be strengthened.

## Acknowledgements

We thank our participants and the staff of Education District 1 and 2 of Hamadan City.

## Conflict of interest statement

No conflict of interest is declared.

## Funding

This study has been supported by Hamadan University of Medical Sciences, Grant (960115134).

## Highlights

- The mean age of WPS initiation was 13.6 (±2.31) yr.
- Educational program based on MTM had effects on participatory dialogue and behavioral confidence (initiation of reducing WPS).
- Educational program based on MTM had effects on emotional transformation and practice for change (maintenance of reducing WPS).
- MTM could provide suitable intervention framework to reduce WPS in male adolescent students.
